# Enhancing methyl parathion degradation by the immobilization of *Burkholderia* sp. isolated from agricultural soils

**DOI:** 10.1002/mbo3.507

**Published:** 2017-07-17

**Authors:** Maikel Gilberto Fernández‐López, Carolina Popoca‐Ursino, Enrique Sánchez‐Salinas, Raunel Tinoco‐Valencia, Jorge Luis Folch‐Mallol, Edgar Dantán‐González, Ma Laura Ortiz‐Hernández

**Affiliations:** ^1^ Centro de Investigación en Biotecnología Universidad Autónoma del Estado de Morelos Cuernavaca Morelos México; ^2^ Centro de Investigación en Dinámica Celular del Instituto de Investigación en Ciencias Básicas y Aplicadas Universidad Autónoma del Estado de Morelos Cuernavaca Morelos México; ^3^ Instituto de Biotecnología Universidad Nacional Autónoma de México Cuernavaca Morelos México

**Keywords:** *Burkholderia* sp., cell immobilization, methyl parathion, *p*‐nitrophenol

## Abstract

Organophosphate pesticides are of great interest for research because they are currently the most commonly used pesticides. In this study, a bacterial strain capable of completely degrading methyl parathion (MP) was isolated from agricultural soils in central Mexico. This strain was designated strain S5‐2 and was identified as *Burkholderia cenocepacia*. To increase degradation yields, cells were immobilized on three different supports: powdered zeolite and *Opuntia* sp. and *Agave* sp. fibers. The results indicated a significant increase in MP hydrolysis and *p*‐nitrophenol (PNP) degradation with immobilized cells compared to free cell cultures. Furthermore, immobilized cells were capable of withstanding and degrading higher concentrations of PNP compared to cell suspension cultures. The cell viability in the free cell cultures, as well as PNP degradation, was affected at concentrations greater than 25 mg/L. In contrast, cells immobilized on *Opuntia* sp. and *Agave* sp. fibers completely degraded PNP at concentrations of 100 mg/L. To verify that MP solution toxicity was decreased by *B*. *cenocepacia* strain S5‐2 via pesticide degradation, we measured the acetylcholinesterase activity, both before and after treatment with bacteria. The results demonstrate that the activity of acetylcholinesterase was unaffected after MP degradation by bacteria.

## 1 INTRODUCTION

Organophosphate pesticides (OPs) are a group of compounds derived from phosphoric acid that are widely used in agriculture as insecticides (Ortiz‐Hernández & Sánchez‐Salinas, 2010). OPs irreversibly inhibit the enzyme acetylcholinesterase, causing damage to the nervous system, respiratory paralysis, and death (Sogorb & Vilanova, 2002). Due to its low specificity, a wide range of organisms are affected by these pesticides (Ali et al., 2011). Furthermore, because of its high solubility in lipids, OPs accumulate in the food chain, thereby increasing the risk of toxicity in humans (Wang et al., 2008).

The accumulation of these pesticides worsens environmental problems associated with organophosphate pesticides, such as when they reach their expiration date and are used under unfavorable conditions and with inefficient regulation. To overcome this problem, physical and chemical techniques have been developed to degrade pesticides partially or completely (Basel Convention, 2014). Due to their high cost and the waste generated after the process, however, these methods remain inefficient. For this reason, the use of microorganisms isolated from contaminated sites that can degrade the OPs has gained greater acceptance, particularly due to microorganisms’ ability to perform complete mineralization of the pesticide without causing environmental damage.

Methyl parathion (O,O‐dimethyl‐O‐4‐*p*‐nitrophenyl phosphorothioate) is an OP. In Mexico, MP is one of the most commonly used pesticides and has been used extensively for insect control with crops, including avocados, rice, onions, peaches, spinach, and strawberries. Although the use of MP is legal, its trade has recently been restricted (Torres, Ramos, Avila, & Ortiz, 2012), and it can be found in soil and water, as well as in waste. It is considered extremely dangerous and is included in the Ia Category of the World Health Organization (WHO, 2009). This pesticide poorly inhibits acetylcholinesterase activity; however, it is metabolically activated by cytochrome *P*_450_ to produce oxons that are very potent inhibitors of this enzyme (Sultatos, 2006). Initially, interaction between the pesticide and enzyme is reversible, but over time, a process called “aging” occurs, which results in the formation of a covalent bond that is much more stable. This process results from the elimination of one of the alkyl side chains of the phosphate group, leaving the hydroxyl group, which avoids regeneration of the active site of the enzyme (Wilson, Colman, & Sutton, 2001).

The first report of a microorganism capable of degrading an organophosphorus compound dates from 1973, when the strain *Flavobacterium* sp. was isolated from a rice field in the Philippines (Sethunathan & Yoshida, 1973). This bacterium was subsequently reclassified as *Sphingobium fuliginis* by Kawahara, Tanaka, Yoon, and Yokota (2010). Although the hydrolytic activity of these bacteria on MP is high, their ability to degrade the PNP generated after MP hydrolysis is limited (Adhya, Barik, & Sethunathan, 1981). PNP is less toxic to animals than MP; however, it is resistant to degradation and is toxic to many microorganisms. There are multiple reports of microorganisms that can hydrolyze MP; however, PNP generated during the hydrolysis of MP may be a limiting factor in the development of biotechnological tools for pesticide degradation. Therefore, a bacterium that can hydrolyze MP and simultaneously degrade PNP is a valuable tool for the biological treatment of MP waste and/or for the remediation of sites contaminated with this pesticide.

Cell immobilization was previously used to make pesticide biodegradation processes more efficient (Kadakol, Kamanavalli, & Shouche, 2011; Yañez‐Ocampo, Sanchez‐Salinas, Jimenez‐Tobon, Penninckx, & Ortiz‐Hernández, 2009; Yáñez‐Ocampo, Sánchez‐Salinas, & Ortiz‐Hernández, 2011). This provides multiple advantages over conventional biological systems that use free cells. These benefits include high cell concentrations, reuse of cells, the elimination of “cell wash” problems at a high dilution rate, high productivity yields (Cassidy, Lee, & Trevors, 1996; Chung, Tseng, & Juang, 2003), and the ability to maintain catalytic activity for long time periods (Martín et al., 2000).

In this study, a bacterial strain that can completely degrade MP was isolated from agricultural soils. The cells were immobilized on *Opuntia* sp., *Agave*, and zeolite. These three supports are waste materials that are abundant in the region and can be used for biotechnological purposes. The results obtained using cells in suspension and immobilized in three different supports are reported. We also conducted different analyses to test the toxicity of the resulting solutions after treatment with the isolated bacteria.

## 2 MATERIALS AND METHODS

### 2.1 Reagents and culture media

Methyl parathion (99% purity) and PNP (99% purity) were purchased from Chem Service (http://web1.chemservice.com). MP was prepared in HPLC grade methanol and added to the culture media at different concentrations as indicated. To prepare the preinoculum, tryptone soy broth was used (Bioxon Becton Dickinson of Mexico, Mexico State, Mexico), and for the biodegradation experiments, a modified mineral salt medium (MSM), originally described by O'Reilly and Crawford (1989), was used with the following composition: 0.82 g/L K_2_HPO_4_, 0.19 g/L KH_2_PO_4_, 0.20 g/L MgSO_4_·7H_2_O, 2.0 g/L KNO_3_, 0.99 g/L (NH_4_)2SO_4_, and 2 ml/L of a trace element solution with a composition of 2.8 g/L H_3_BO_3_, 2.55 g/L MnSO_4_·H_2_O, 0.17 g/L CuSO_4_·5H_2_O, 2.43 g/L CoCl_2_·6 H_2_O, and 0.25 g/L ZnSO_4_·7H_2_O. High‐performance liquid chromatography (HPLC) grade methanol (Mallinckrodt Baker, Inc., Phillipsburg, NJ) was used to inject samples into a HPLC. All other chemicals were of reagent grade and were obtained from J.T. Baker, Mexico City.

### 2.2 Isolation and selection of the strain

Soil samples were taken from a commercial cornfield in central Mexico (latitude 18° 58′26″ N and longitude 99° 07′05″ W). The soil was a vertisol type and was collected at 10 cm below the soil surface. Sterile Erlenmeyer flasks were used to store and transport samples at 4°C until isolation of the bacteria. After reaching the laboratory, soil samples were enriched with MP by adding aliquots every week for 30 days, reaching a concentration of 200 mg/L. The enriched soil samples were incubated in the dark at 30°C (Goda, Elsayed, Khodair, El‐Sayed, & Mohamed, 2010). Subsequently, 1 g of each sample was collected and inoculated into 50 ml of MSM supplemented with MP (50 mg/L) and incubated for 7 days at 30°C at 140 rpm. An aliquot of the culture medium was used to inoculate another flask of fresh MSM with MP (50 mg/L), leaving it to grow for 7 days under the same conditions. After three subsequent repetitions, an aliquot of 100 μl was taken and seeded in Petri dishes with tryptone soy agar supplemented with 50 mg/L of the pesticide. These were incubated at 30°C for the necessary time until bacterial colonies were visible.

The colonies that showed yellow halos around of them (indicative of MP hydrolysis to PNP) were selected. The selected strains were cultured in MSM supplemented with MP, and the production of PNP and its subsequent degradation was monitored spectrophotometrically at 410 nm. Those strains that could perform both processes (MP hydrolysis and PNP degradation) were selected. The strain that showed the greatest efficiency degradation of both MP and PNP was selected and this colony was labeled as S5‐2.

### 2.3 Characterization of the selected strain

The isolate was identified by biochemical tests (Voges–Proskauer, methyl red, motility–indole–ornithine, citrate Simons, catalase, and Gram stain) and 16S rRNA gene analysis. Genomic DNA extraction was performed using a kit from Axygen and this was used as substrate for the amplification of the 16S rRNA gene using the universal oligonucleotides L1041 5′‐CGGTGTGTACAAGACCC‐3′ and PROK63FW 5′‐CAGGCCTAACACATGCAAGTC‐3′ (Felske, Engelen, Nübel, & Backhaus, 1996). The amplification products were purified using the High Pure PCR Product Purification (Roche) kit, and they were subsequently sequenced and compared with GenBank sequences. Phylogenetic analysis of the 16S rRNA gene sequences was performed using MUSCLE version 3.7 (Dereeper et al., 2008; Dereeper, Audic, Claverie, & Blanc, 2010). Phylogenetic trees were constructed with the MEGA 6.06 program with the neighbor‐joining method (Tamura, Stecher, Peterson, Filipski, & Kumar, 2013) and 1,000 bootstrap replicates.

### 2.4 Inoculum

The selected strain was inoculated into the tryptone soy broth supplemented with MP to a final concentration of 50 mg/L for 18 hr at 30°C with agitation at 120 rpm. Subsequently, the biomass was collected by centrifugation and washed twice with a sterile solution of 0.8% NaCl (w/v) and resuspended in the same solution. This suspension was adjusted to an optical density of approximately 0.5 at 600 nm and the resulting suspension was used as an inoculum for MP degradation tests. In all experiments, 1% v/v inoculum was added to the culture medium and MP to a concentration of 50 mg/L.

### 2.5 Growth and degradation kinetics using free cells

For free cell cultures, growth and degradation kinetics were assessed. Sterile Erlenmeyer flasks of 125 ml were supplemented with MSM and MP at a final concentration of 50 mg/L. A 1% (v/v) inoculum with biomass prepared as described above was added. All flasks were incubated on a shaking platform for 24 hr at 120 rpm and 30°C. The removal of MP, generation and subsequent PNP degradation, and the growth of bacteria by viable count were measured immediately after inoculation and at different time intervals. MSM with MP and without inoculum and MSM with inoculum and without MP were used as controls. All treatments were performed in triplicate.

### 2.6 Material used as a support for cell immobilization

For cell immobilization, the following materials were used as supports:

#### 2.6.1 *Opuntia* sp. fibers

*Opuntia* sp. is a genus belonging to the *Cactaceae* family, growing in fields throughout central and northern Mexico and the southwestern United States. The plant material was collected in the central region of Mexico and was left to dry after collection. A porous, strong structure that makes it favorable for cell immobilization is then formed. These dry leaves of *Opuntia* sp. were cut into small pieces (approximately 2 cm^2^), washed with water, and dried in the oven at 50°C. The composition (%) of the *Opuntia* sp. fibers is as follows: structural carbohydrates, 36.3 ± 1.1; cellulose, 13.1 ± 0.7; hemicellulose, 18.5 ± 0.7; lignin, 12.3 ± 1.1; protein, 7.4 ± 0.3; extractives, 25 ± 0.9; and ash, 23.7 ± 0.1 (Yang et al., 2015).

#### 2.6.2 *Agave* sp. fibers

*Agave* sp. is a plant widely used in Mexico for tequila production. During tequila production, the leaves of the plant are discarded and large amounts of waste‐like fibers are generated. These fibers are highly resistant and can be used for cell immobilization. *Agave* sp. fibers were autoclaved at 121°C for 15 min. Next, the fibers were macerated to separate the fibers and washed with water. The fibers were left overnight in acetone, washed with water, and allowed to dry in an oven at 50°C. The composition (%) of *Agave* sp. fibers is as follows: structural carbohydrates, 43.8 ± 1.3; cellulose, 26.0 ± 1.2; hemicellulose, 22.8 ± 1.2; lignin 13.8 ± 1.3; protein, 2.4 ± 0.1; extractives, 29.0 ± 1.2; and ash, 6.0 ± 0.1 (Yang et al., 2015).

#### 2.6.3 Powdered zeolite

Zeolites are microporous crystalline solids with well‐defined structures and shape‐selective properties. They are widely used in molecular adsorption (Datta, Christena, & Rajaram, 2012) as well as in adsorption and separation processes, environmental pollution control, petrochemical processes, fine chemical synthesis, biochemistry for the synthesis of nanocomposite materials, and cell immobilization (Tope, Srinivas, Kulkarni, & Jamil, 2001). Among a wide range of adsorbents available, the unique physical and chemical properties of natural zeolites, particularly their high porosity and large surface area, have made them useful for the immobilization of microorganisms (Shindo, Takata, Taguchi, & Yoshimura, 2001).

The zeolite used in this study is the waste of an industrial process in which it is used as a carrier for the manufacture of food supplements. This material is ground and sieved in a 500 mesh, resulting in particles with a size of approximately 25 microns, which provides a large surface area. The X‐ray analysis revealed the following elemental composition (%): C, 9.23; O, 44.87; Na, 3.48; Al, 6.15; Si, 32.18; and Cl, 4.09. Before being introduced into the flasks for cell immobilization, the zeolite was washed with abundant distilled water.

### 2.7 Growth and degradation kinetics using immobilized cells

Supports were added at 2% (w/v) to tryptone soy broth medium and sterilized by autoclaving for 15 min at 121°C twice with an intermediate repose of 24 hr. Next, the media was inoculated with the S5‐2 strain and incubated for 24 hr at 30°C and 100 rpm to allow colonization of the bacteria on the supports. We next proceeded to wash the supports twice with 0.8% NaCl solution (w/v) to eliminate free cells by centrifugation at low speed (1250 g for 5 min). The mineral medium described above supplemented with MP as a carbon source was added to the supports. To measure the MP and PNP concentrations, samples at regular intervals were taken and analyzed by HPLC. To evaluate bacterial survival immobilized on the supports, the viable count was determined for each treatment, collecting samples weekly for 21 days. The results are reported as CFU/*g*_support_. We also report the percentage of survival of bacteria, which was calculated from the CFU/*g*_support_ values obtained at the end of the kinetics, with respect to the initial values.

To desorb the pesticide adsorbed by the zeolite, *Opuntia* sp., and *Agave* sp., the uninoculated controls (MSM with MP) were used. After 24 hr incubation, the MSM was removed and the supports were washed twice with 0.8% NaCl solution (w/v). After that, ethyl acetate was added to the supports and they were shaken at 250 rpm for 1 hr. Then, they were sonicated for 30 min and 1 ml of the supernatant was removed and dried. Samples were suspended in a methanol–water mixture and analyzed by HPLC.

### 2.8 Toxicity tests

Toxicity tests were performed in two ways to evaluate distinct aspects of PNP effects at different levels. It has been reported that PNP causes acute toxicity to biological systems (Bhushan, Chauhan, Samanta, & Jain, 2000), so we decided to test its effect on the growth of the strain at several PNP concentrations using free and immobilized cells. It is also well documented that organophosphate pesticides inhibit the activity of acetylcholinesterase, and for this reason, we measured the activity of this enzyme before and after treatment of media containing MP.

#### 2.8.1 Toxicity test of PNP on the isolated strain

To measure the PNP effect on the isolated strain, the same methodology described in the previous section was followed; however, the MP was replaced by PNP and different concentrations were tested (25, 50, 100, 200, and 300 mg/L). Viability was measured by counting viable cells and PNP concentration measurement at the initial time and 24 hr after incubation.

#### 2.8.2 Acetylcholinesterase inhibition by MP added to the culture media before and after treatment with the isolated strain

To determine whether treatment with the bacterial strain reduces acetylcholinesterase inhibition by methyl parathion, we measured the acetylcholinesterase activity both before and after pesticide treatment. This measurement was performed according to the methodology developed by Ellman, Courtney, Andres, and Featherstone (1961) with several modifications. The S9 fraction of rat liver was added to the reaction mixture to oxidize the MP by cytochrome *P*_450_ and thus become the oxon derivative active on acetylcholinesterase (Sultatos, 2006). To use this fraction, a primarily S9 mixture was prepared as follows: distilled water, 375 μl; phosphate buffer 0.2 mol/L, 500 μl; glucose‐6‐phosphate 1 M, 5 μl; NADP 0.1 mol/L, 40 μl; KCl 1.65 mol/L, 20 μl; MgCl_2_ 0.4 mol/L, 20 μl; and S9 fraction of rat liver, 40 μl (Ames, McCann, & Yamasaki, 1975).

A volume of 475 μl of MSM with pesticide, before and after each treatment, was mixed with 15 μl of an acetylcholinesterase solution (5 U/ml) and 500 μl of S9 mixture. Next, the mixture was incubated at 37°C and 450 rpm. For the enzymatic activity measurement, 150 μl of the reaction mixture was collected every 30 min and placed in a 96‐well plate and mixed with 150 μl of phosphate buffer (pH = 8), acetylthiocholine iodide and DTNB in a proportion of 150:2:10. The assay was performed in triplicate, and the results were plotted as residual enzymatic activity versus time of exposure between acetylcholinesterase and methyl parathion. The controls used were noninoculated mineral medium with or without methyl parathion. One unit (U) of acetylcholinesterase activity catalyzes the release of 1 μmol of nitrobenzoate per minute at 25°C. Specific activity is expressed as units of enzyme activity per mg of protein.

### 2.9 Analytical methods

To quantify the MP and PNP concentration, 1 ml of sample from each flask was collected at different time intervals and extracted with 1 ml of ethyl acetate. The organic phase was recovered and filtered through a glass funnel packed with glass wool and anhydrous sodium sulfate. The extract was collected in amber vials and the procedure was repeated three times by adding 1 ml of ethyl acetate each time, then mixing and recovering the organic phase (Ortiz‐Hernández & Sánchez‐Salinas, 2010). Finally, the contents of the vials were allowed to dry and 1 ml of 50% HPLC grade methanol was added. Samples were analyzed by HPLC on a C18 column (3.5 μm; 4.6 × 100 mm, Waters, Model XTerra^®^ MS), with a flow rate of 0.8 ml/min in a 60:40 methanol–water system, at 30°C, and readings were recorded at 270 nm.

### 2.10 Preparation of immobilizing material for observation in a scanning electron microscope

Cell immobilization was confirmed by scanning electron microscopy field emission (Hitachi S‐5500). The material used for immobilization was dried for 48 hr at 70°C. Samples were observed directly on the microscope.

### 2.11 Statistical analysis

The final concentrations of removed MP were transformed using an angular transformation (arcsine) and were analyzed using the general linear models procedure (SAS, PROC GLM) as an analysis of variance. Post hoc analysis of differences in means was conducted with the Tukey's test (α = 0.05).

## 3 RESULTS

### 3.1 Isolation and identification of S5‐2 strain

In this study, it was possible to isolate a strain, designated S5‐2, that was capable of hydrolyzing MP, and using PNP as a sole carbon source for growth in the mineral medium. This strain is a Gram‐negative bacillus, with responses in the biochemical tests that were negative for methyl red and Voges–Proskauer, and positive for motility, indole, ornithine, Simmons citrate, and catalase. The results of the biochemical tests and phylogenetic analysis allowed this strain to be included in the *Burkholderia cepacia* complex, with its nearest neighbor *B. cenocepacia* CEIB S5‐1 (details of data submission can be found at DDBJ/EMBL/GenBank under the accession no. JTLT00000000) (Martínez‐Ocampo et al., 2015).

### 3.2 Growth kinetics and pesticide degradation in suspension cultures

**Figure 1:**
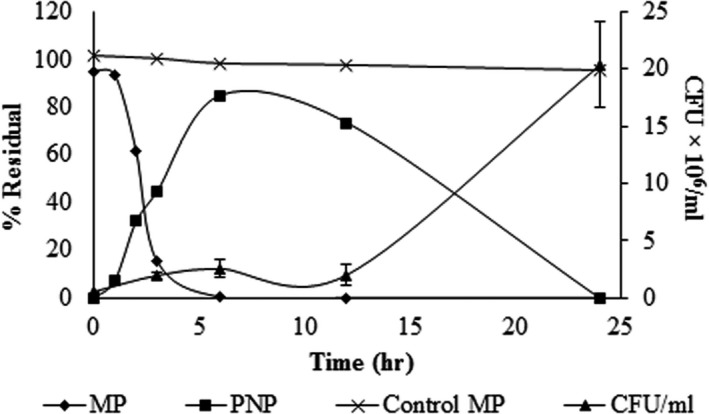
Growth kinetics of *Burkholderia* sp. strain S5‐2 and MP mineralization. MP: residual methyl parathion (%), with respect to the initial concentration; PNP: residual PNP (%) with respect to the concentration generated by the MP hydrolysis; Control MP: methyl parathion (%) with respect to the initial concentration in the noninoculated MSM

Growth kinetics and pesticide degradation in suspension cultures showed that MP was completely hydrolyzed during the first 6 hr of culture (Figure 1). The PNP concentration in the culture medium increased while MP hydrolysis occurred, and its higher concentration occurred when the entire MP added to the medium was hydrolyzed. Subsequently, the newly formed PNP concentration decreased to zero. Biomass growth was associated with the degradation of PNP, suggesting that cells used this compound as a source of carbon and energy.

### 3.3 Pesticide degradation kinetics with immobilized cells

**Figure 2:**
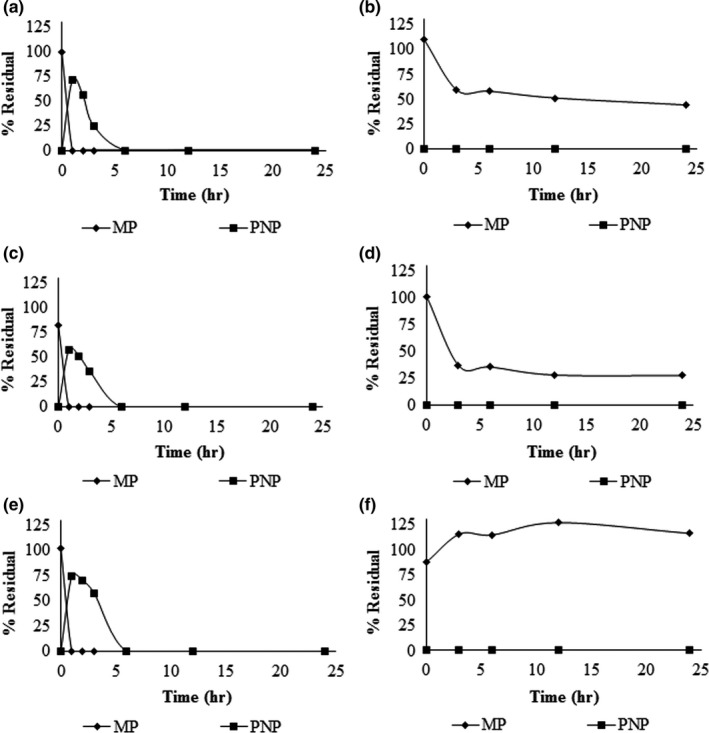
MP and PNP degradation kinetics with immobilized cells. (a) MP degradation using *Opuntia* sp. fibers with cells. (b) *Opuntia* sp. fibers without cells. (c) MP degradation with immobilized cells on *Agave* sp. (d) *Agave* sp. without cells. The same conditions are shown in (e) and (f), but using zeolite. In controls without cells (b, d, and f), PNP was not detected because the MP hydrolysis did not occur via abiotic factors (PNP was not added to the culture media). All supports were added at 2% w/v to the culture medium

At the beginning of the degradation tests with immobilized cells, the biomass was 600 × 10^6^ CFU/*g*_support_ for *Opuntia* sp., 730 × 10^6^ CFU/*g*_support_ for *Agave* sp., and 99 × 10^6^ CFU/*g*_support_ for zeolite. Each treatment with immobilized cells showed similar behavior in the degradation process and they were superior to suspension cultures. Complete removal of MP was achieved in less than 1 hr, and the PNP degradation generated during the hydrolysis occurred in less time compared to cell‐free cultures. By contrast, when the MP was in contact with the materials used as a support, without immobilized cells, the MP concentration in the culture medium decreased, likely due to a MP adsorption process on the fibers used (Figure 2).

Our results show that after the MP is added to the culture medium with the respective fibers used, this compound is adsorbed into the fibers between 50% and 70% and the remainder of the MP remains in the culture medium, which is hydrolyzed by bacteria. It is necessary to clarify that in this experiment, the PNP was not added to the culture medium; therefore, this compound is not generated due to the absence of the strain (Figure 2). To verify the MP desorption of the fibers, however, we found that the adsorbed amount corresponds to 12.4% and 29.1% of the pesticide with respect to its initial concentration in supports *Opuntia* and *Agave*, respectively. Although not all the pesticide was desorbed by the method proposed, the results show that PNP is not generated, indicating no abiotic degradation of the pesticide. Therefore, the removal of the MP in the culture medium occurs via a combined action between its adsorption on the supports and degradation in the culture medium by the strain.

**Figure 3:**
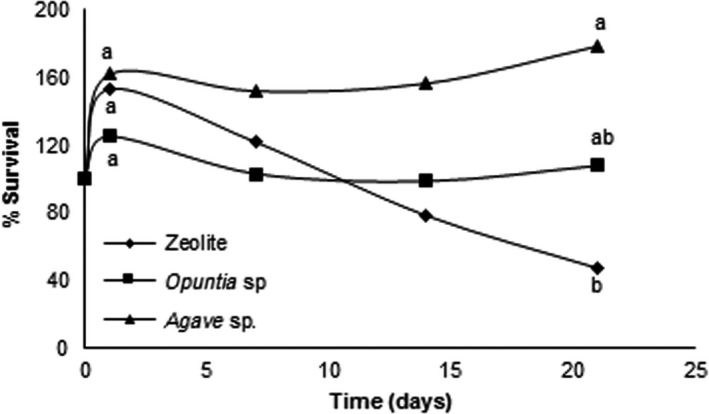
Survival of the *Burkholderia* sp. strain S5‐2 immobilized on *Opuntia* sp., *Agave* sp., and zeolite. Percentage survival of bacteria was calculated from the CFU/*g*_support_ values of at the end of the kinetics, with respect to the initial values. The same letters are not significantly different (α = 0.05). All supports were added at 2% w/v to the culture medium

To evaluate strain stability, viability for each treatment was determined for 21 days. The results showed a significant decrease in survival for the cells immobilized on powdered zeolite. For *Opuntia* sp. and *Agave* sp. fibers, there was no significant loss of viability (Figure 3).

### 3.4 Evidence of immobilization by scanning electron microscopy

**Figure 4:**
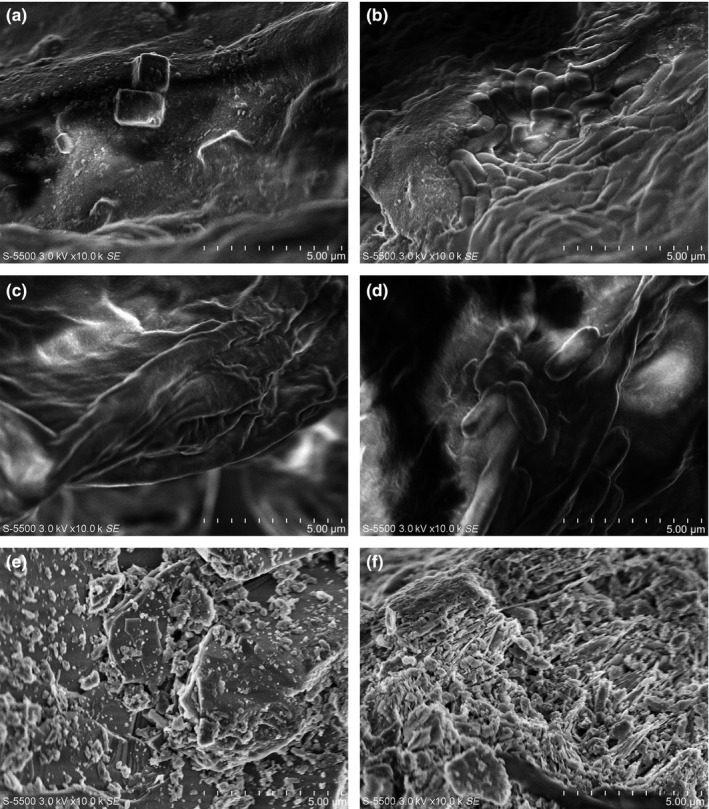
Scanning electron micrographs at 10,000× of the supports used for cell immobilization. (a) Control *Opuntia* sp. without biomass. (b) *Burkholderia* sp. strain S5‐2 immobilized on *Opuntia* sp. (c) Control *Agave* sp. (d) *Agave* sp. fibers with immobilized cells. (e) Control zeolite. (f) Zeolite with immobilized cells

The bacterial ability for biofilm formation on surfaces is a very useful tool for biotechnology. To confirm that the *Burkholderia* sp. strain S5‐2 can support colonization, scanning electron microscopy of the three supports used in this paper was performed. The results showed that bacteria are trapped on the surface of *Opuntia* sp. and *Agave* sp. fibers after incubation for 24 hr. In zeolite, unimmobilized cells on the surface were observed (Figure 4).

### 3.5 Toxicity tests

#### 3.5.1 *p*‐Nitrophenol toxicity for the strain

MP hydrolysis yields PNP, and this compound is toxic to most bacteria. This could be a limiting factor if these bacteria are used to degrade waste with high MP concentrations. We performed growth kinetics using MSM and PNP at different concentrations. The results of these experiments indicate that the *Burkholderia* sp. strain S5‐2 used PNP as a carbon source. Figure 6a shows the effect of different concentrations of PNP on the free cell growth of the *Burkholderia* sp. strain S5‐2, as well as PNP removal in the culture medium. The lowest concentration of PNP tested allowed microbial growth and degradation of this compound. Concentrations above 25 mg/L completely inhibited bacterial growth, however, confirming the toxic effect of PNP on the viability of the isolated strain.

**Figure 5:**
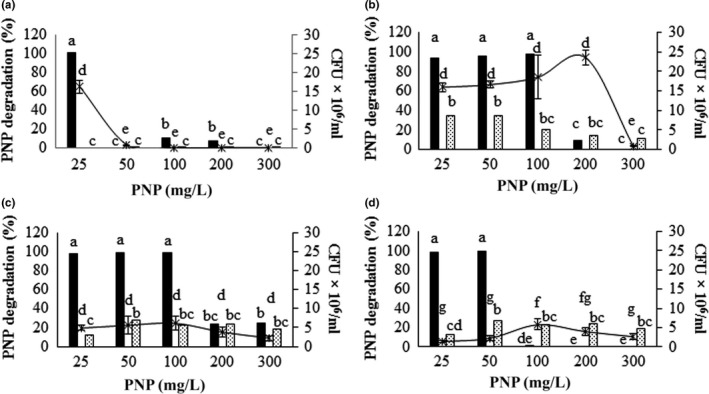
Effect of different concentrations of PNP on the process of degradation of this compound and the viability the of the S5‐2 strain. (a) Cells in suspension. (b, c, d) Cells immobilized in *Opuntia* sp., *Agave* sp., and zeolite, respectively. The PNP (▪) and UFC/ml of *Burkholderia cenocepacia* S5‐2 strain (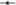) was determined after 24 hr of treatment in mineral medium supplemented with PNP and inoculated with strain S5‐2. As a control, the same medium is used uninoculated (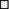). Different letters indicate significant differences (α = 0.05). All supports were added at 2% w/v to the culture medium

PNP also showed an inhibitory effect on growth in immobilized cells, but to a lesser extent. Immobilized cells on *Opuntia* sp. fibers completely degraded PNP at concentrations of 100 mg/L without affecting their viability (Figure 5b). At 200 mg/L, maximum growth was evident; however, only 17% of the PNP was degraded. When *Agave* sp. fibers with the immobilized cells were incubated with different concentrations of PNP, a decrease between 60% and 80% of immobilized cells was observed compared to values reported at the beginning of the experiment (Figure 5c); however, all the PNP is degraded at a concentration of 100 mg/L. The amount of immobilized cells on zeolite was lower compared to supports of vegetable origin, but the PNP was degraded up to a concentration of 50 mg/L; concentrations above this limit cause a decrease in viability and degradation (Figure 5d).

#### 3.5.2 Acetylcholinesterase inhibition by culture media before and after treatment with the isolated strain

To assess the MP toxicity before and after treatment with the isolated strain, the acetylcholinesterase residual activity was measured at different time intervals. Free or immobilized cells of the strain were cultured in MSM and MP, and after the treatment (see below), the culture medium was used to test its capacity to inhibit acetylcholinesterase activity at different time intervals. The results obtained using a control mixture of acetylcholinesterase, S9 mix, and phosphate buffer, pH 8, showed that in 30 min, the enzyme had no significant loss of activity by abiotic factors (data not shown). This incubation time was sufficient to achieve significant enzyme inhibition by the pesticide. This result led to the selection of this time as the most suitable for this trial.

**Figure 6:**
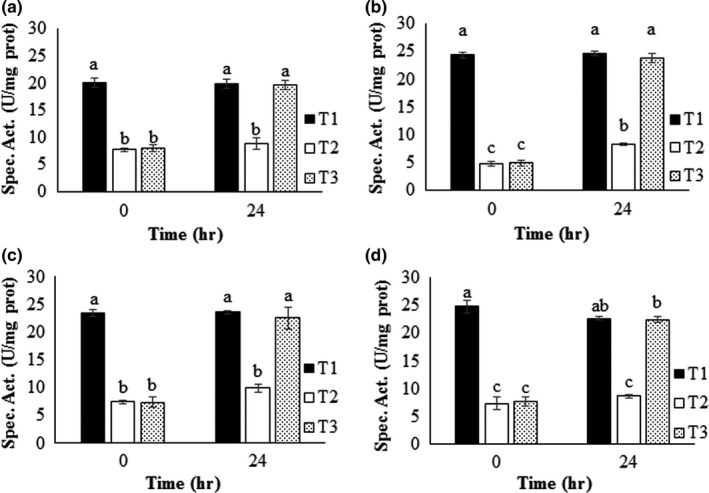
Toxicity test of the resulting solutions obtained before and after each treatment. (a) Cells in suspension. (b, c, d) Cells immobilized on *Opuntia* sp., *Agave* sp., and zeolite, respectively. T1: control of MSM. T2: control MSM with MP (50 mg/L). T3: MSM with MP (50 mg/L) inoculated with *Burkholderia* sp. strain S5‐2. Each treatment was contacted with acetylcholinesterase and residual enzyme activity after 30 min of inactivation was determined. The enzyme is inactivated by MP except those treatments where pesticide degradation occurs by strain S5‐2. Different letters indicate significant differences (α = 0.05)

The results showed that after 24 hr of culture with the isolated strain in MP, the resulting medium lost its capacity to inhibit the acetylcholinesterase activity, indicating that the MP was completely transformed. Therefore, this treatment is promising for detoxifying solutions containing MP (Figure 6). In addition, control media with supports only do not have negative effects on the activity of acetylcholinesterase. There were differences in the MSM with MP treatment without bacteria, demonstrating that the acetylcholinesterase activity remained low (α=0.05). In contrast, the acetylcholinesterase assays using the culture media before and after each treatment with free and immobilized cells with MSM and without MP (control) showed that the acetylcholinesterase activity was not lost during the incubation time (Figure 6).

## 4 DISCUSSION

A bacterial strain with ability to hydrolyze MP, and mineralized PNP, was isolated from contaminated soil. Biochemical and molecular tests allowed for the inclusion of this strain in the *B. cepacia* complex. There are reports of the genus *Burkholderia* that have genes involved in the degradation of organophosphorus pesticides (Chino‐Flores et al., 2012); however, the ability of this particular strain to degrade PNP, which is toxic to microorganisms, makes it a useful tool in biotechnology for bioremediation purposes. Nitroaromatic compounds are released into the environment because they have wide use as dyes, pesticides, plasticizers, explosives, and solvents (Spain & Gibson, 1991). Aromatic compounds are relatively resistant to biomineralization due to enhanced stability caused by the resonance energy of the aromatic ring (Ismail & Gescher, 2012). The *Burkholderia* sp. strain S5‐2 can degrade an aromatic compound such as PNP, however, which begs the question: what other aromatic pollutants will this strain be able to mineralize?

There is a report in *B. cepacia* of the *mpd* gene coding for methyl parathion hydrolase, which is responsible for the hydrolysis of MP (Ekkhunnatham, Jongsareejit, Yamkunthong, & Wichitwechkarn, 2012). Vikram et al. (2012) found in the *Burkholderia* strain SJ98 genes encoding monooxygenases responsible for the degradation of aromatic compounds. However, we do not have any report of *B. cenocepacia* that can hydrolyze the MP and degrade PNP. We do not know the molecular mechanisms by which the *B. cenocepacia* strain S5‐2 hydrolyzes the MP and degrades the PNP, however, opening the doors for future investigations to solve this question.

PNP and other nitroaromatics have been reported to be highly toxic to most microorganisms, thus limiting its degradation at high concentrations (Samuel, Sivaramakrishna, & Mehta, 2014). It has been reported, however, that some bacteria such as *Pseudomonas*,* Arthrobacter*,* Flavobacterium*,* Nocardia*, and *Moraxella* can metabolize PNP at low concentrations (Kulkarni & Chaudhari, 2007; Leung, Moore, Lee, & Trevors, 2005; Zhang et al., 2009). More recently, attention has turned to the isolation of bacteria capable of degrading PNP at high concentrations (in the order of 100 mg/L) that are toxic to previously studied strains (Ray, Oubelli, & Löser, 1999).

In this work, when the degradation tests were performed using suspended cells, a slight delay in *B. cenocepacia* strain S5‐2 growth was observed during the first 12 hr, which is associated with the toxic effect of PNP. The gap between the PNP decrease and biomass growth may occur because PNP must be transformed by one of the two oxidative pathways described in previous reports, hydroquinone and hydroxyquinol (Kadiyala & Spain, 1998; Kitagawa, Kimura, & Kamagata, 2004; Spain & Gibson, 1991; Zhang et al., 2012). Both pathways transform the PNP into β‐ketoadipate, which enters the tricarboxylic acid cycle, allowing for the production of energy and growth. Thereafter, the bacterial growth was exponential.

There are few reports of isolates that can perform both MP hydrolysis and PNP degradation. *Burkholderia* sp. strain S5‐2 can perform both processes, however, suggesting a total mineralization of MP, although their growth is initially limited by PNP toxicity. The toxic effect of PNP was evident when the strain was grown with different concentrations of this compound, which made it necessary to establish new strategies to overcome this drawback using cell immobilization. Cell immobilization offers many advantages addressed earlier in this study; however, an appropriate material for this process, including characteristics such as being economical, inert, and affordable, is difficult to find. The different supports proposed in this study comply with these characteristics. Thus far, we have not found reports for the supports used in this study for the biological removal of organophosphate pesticides, although related studies were available. For example, zeolite was studied to immobilize the *Pseudomonas* sp. strain ADP for atrazine degradation (Stelting, Burns, Sunna, Visnovsky, & Bunt, 2012). This research showed that this bacterial species remained viable for 10 weeks at 25°C. Another study reported that *Agave* sp. fiber was used to immobilize fungi (*Trametes versicolor* and *Pleurotus ostreatus*) to study the degradation of the dye triphenylmethane basic green 4. Immobilized fungal cultures on *Agave* fiber without addition of cosubstrate was selected as the best option, resulting in decoloration efficiencies of 99.3% (Castillo, 2010).

There are multiple reports showing that the immobilization of cells promotes xenobiotic degradation processes. Tallur, Mulla, Megadi, Talwar, and Ninnekar (2015) investigated the degradation of cypermethrin by immobilized cells of *Micrococcus* sp. strain CPN 1 in various supports such as polyurethane foam, polyacrylamide, sodium alginate, and agar. The study revealed that *Micrococcus* sp. strain CPN 1 immobilized cells were more effective for the degradation of cypermethrin at higher concentrations than freely suspended cells. In another study, Briceño, Fuentes, Rubilar, Jorquera, and Tortella (2013) evaluated diazinon removal achieved by free actinobacteria and *Streptomyces* sp. AC1‐6 immobilized on alginate beads. The study revealed that the immobilized cells exhibited 60% higher diazinon removal compared with the free cells. The immobilized microbial systems have the advantage of an enhanced rate of degradation, tolerance to higher substrate concentrations, and its reusability.

Immobilized cells resisted higher concentrations of PNP compared to suspended cells. Being immobilized in *Agave* sp., however, not only affects cell viability at high concentrations of PNP (≥200 mg/L) but also has a negative effect on the ability of bacteria to adhere to this support, as even at the lowest concentration tested we only found 20%–40% of the cells adhered compared to the control without PNP.

Strain immobilization on the three supports was evaluated by scanning electron microscopy. Immobilized cells on the proposed supports were very efficient in the degradation of MP and no differences were found in the times of degradation. In these treatments, however, the degradation was higher than in free cell cultures. The amount of immobilized cells on zeolite was less than *Opuntia* sp. and *Agave* sp., likely because this support is pulverized and the cells are much more exposed to the environment and turbulence generated during agitation, preventing biofilm formation on the surface of the support. This is likely why immobilized cells were not observed in zeolite by scanning electron microscopy. In addition, the available surface area for immobilization on zeolite is minor compared to supports of vegetable origin. Although the amount of immobilized cells for each treatment differs significantly, the time required to hydrolyze MP and degrade PNP is not different, and this may be because the system reached the maximum degradation rate for the concentration of pesticide used in our experiments.

For the *Opuntia* sp. and *Agave* sp. supports, the amount of adsorbed pesticide was superior to zeolite. The rapid hydrolysis of MP and PNP degradation make it difficult to determine the extent to which the sorption phenomenon affects the degradation process. Nevertheless, the adsorption of the pesticide by the supports may promote the degradation process. As an example, there is a report in which *Acinetobacter venetianus* was immobilized on bagasse for the degradation of *n*‐alkane. The degradation of the alkane was based on both adsorption and biodegradation by immobilized *A. venetianus*, where 93.3%, 77.7%, and 24.0% of tetradecane (400 mg/L) was removed by the immobilized cells, free cells, and bagasse after 36 hr incubation, respectively (Lin, Gan, Chen, & Naidu, 2015). One advantage of cell immobilization is that it keeps cells viable for long periods. We evaluated the survival of cells for 21 days, and the results showed that the cells immobilized on zeolite lose significant viability at 7 days of incubation. For the immobilized cells on plant fibers, no significant loss of viability was evident. This may be due to the extent of nutrient acquisition from the support because there are reports of *Burkholderia* strains with cellulolytic capacities (Bandounas, Wierckx, de Winde, & Ruijssenaars, 2011; Liang, Zhang, Wu, Wu, & Feng, 2014). Thus, strain S5‐2 may slowly be obtaining carbon from the cellulose and hemicellulose present in these supports. Experiments are underway to test the cellulolytic abilities of this strain and test this hypothesis.

The increased degradation by immobilized cells was likely due to the high local cell density on fibers, together with better cell survivability and an increase in catalytic activity. The combined action of these factors allowed *B. cenocepacia* strain S5‐2 to significantly decrease the PNP degradation time, decreasing the toxic effects of this compound on cells. The amount of biomass in the systems of bioremediation has an important positive effect on the degradation of PNP and other toxic compounds and has been reported previously. For example, Zohar, Kviatkovski, and Masaphy (2013) increased the tolerance of *Arthrobacter* sp. 4Hb to high PNP concentrations by increasing the inoculum size. In their system, PNP toxicity at high initial concentrations (200–800 mg/L) could be overcome by using a high level of inoculum. Their results showed that the initial PNP concentration affected the acclimation stage as well as the linear PNP disappearance stage. Increasing the PNP concentration prolonged the acclimation period and reduced the PNP disappearance rate. Saez, Aparicio, Amoroso, and Benimeli (2015) evaluated the influence of acclimation on a free or immobilized *Streptomyces* consortium on lindane degradation in liquid and slurry systems. After the acclimation period, the survival of all members was confirmed, thus demonstrating the consortium stability. Acclimated cultures reached a higher biomass (0.56–0.65 g/L) and lindane removal (40%–97%) than the consortium without acclimation (0.37–0.44 g/L; 33%–87%) when they were cultured in a liquid medium with 20 and 50 mg/L of lindane.

There are no recent reports that provide information on the use of *Agave* sp. and *Opuntia* sp. fibers for pesticides degradation; therefore, the results found in this study propose an opportunity to degrade MP waste, especially in countries where these fibers are cheap and highly available. Moreover, the materials used are nontoxic to cells, inexpensive, and abundant in Mexico, Latin America, the Middle East, and even some European countries. However, there are risks related to the intrinsic pathogenicity of *B. cenocepacia*. The members of the *B. cepacia* complex are considered opportunistic pathogens, and for this reason, our experiments were conducted under controlled conditions in reactors, without releasing living cells into the environment. For the purposes of bioremediation, we suggest studying genes and enzymes responsible for this degradation pathway and expressing them in another vector that offers greater security against such risks. Toxicological tests, however, showed that the effect of the parental material (MP) on acetylcholinesterase is removed by strain S5‐2, but fails to demonstrate the possibility of the media remaining toxic due to other metabolites derived from MP degradation. Considering this possibility, a test to detect metabolites coming from MP degradation was conducted but none of the reported metabolites were found (data not shown), suggesting total MP degradation. These results encourage the safe use of immobilized *Burkholderia* sp. strain S5‐2 as a system for MP degradation under controlled conditions, for example, in a reactor.

A strain belonging to the genus *Burkholderia* sp. that can completely degrade MP was isolated from agricultural soil in central Mexico. The degradation process was compared between free and immobilized cells on *Opuntia* sp. and *Agave* sp. fibers, as well as powdered zeolite. The results showed that the degradation process is superior with immobilized cells because cells remain viable for longer periods, and the strain can withstand higher concentrations of PNP when immobilized. Toxicity tests for acetylcholinesterase activity demonstrated complete detoxification of the pesticide.

## CONFLICT OF INTEREST

None declared.

## References

[mbo3507-bib-0001] Adhya, T. K. , Barik, S. , & Sethunathan, N. (1981). Hydrolysis of selected organophosphorus insecticides by two bacteria isolated from flood soil. The Journal of Applied Bacteriology, 50, 167–172. 7228819 10.1111/j.1365-2672.1981.tb00881.x

[mbo3507-bib-0002] Ali, M. , Naqvi, T. A. , Kanwal, M. , Rasheed, F. , Hameed, A. , & Ahmed, S. (2011). Detection of the organophosphate degrading gene opdA in the newly isolated bacterial strain *Bacillus pumilus* W1. Annals of Microbiology, 62(1), 233–239.

[mbo3507-bib-0003] Ames, B. M. , McCann, J. , & Yamasaki, E. (1975). Methods for detecting carcinogens and mutagens with Salmonella/mammalian microsomes mutagenicity test. Mutation Research/Environmental Mutagenesis and Related Subjects, 31, 347–364. 10.1016/0165-1161(75)90046-1 768755

[mbo3507-bib-0004] Bandounas, L. , Wierckx, N. J. , de Winde, J. H. , & Ruijssenaars, H. J. (2011). Isolation and characterization of novel bacterial strains exhibiting ligninolytic potential. BMC Biotechnology, 11(1), 1–11. 21995752 10.1186/1472-6750-11-94 PMC3212925

[mbo3507-bib-0005] Basel Convention . (2014). Updated general technical guidelines for the environmentally sound management of wastes consisting of, containing or contaminated with persistent organic pollutants (POPs). Retrieved from: http://www.basel.int/Implementation/Publications/TechnicalGuidelines/tabid/2362/Default.aspx [Accessed April 2017].

[mbo3507-bib-0006] Bhushan, B. , Chauhan, A. , Samanta, S. K. , & Jain, R. K. (2000). Kinetics of biodegradation of p‐nitrophenol by different bacteria. Biochemical and Biophysical Research Communications, 274, 626–630. 10924328 10.1006/bbrc.2000.3193

[mbo3507-bib-0007] Briceño, G. , Fuentes, M. S. , Rubilar, O. , Jorquera, M. , & Tortella, G. (2013). Removal of the insecticide diazinon from liquid media by free and immobilized *Streptomyces* sp. isolated from agricultural soil. Journal of Basic Microbiology, 53, 1–10. 24338785 10.1002/jobm.201300576

[mbo3507-bib-0008] Cassidy, M. B. , Lee, H. , & Trevors, J. T. (1996). Environmental applications of immobilized microbial cells: A review. Journal of Industrial Microbiology, 16(2), 79–101.

[mbo3507-bib-0009] Castillo, L. C . (2010). Remoción del colorante trifenilmetánico verde básico 4 por cultivos mixtos microbianos usando agave tequilero (Agave tequilana weber) como bioportador. Thesis for the degree of master of Science Chemical‐Biological, Instituto Politécnico Nacional, Escuela Nacional de Ciencias Biológicas, Mexico D.F.

[mbo3507-bib-0010] Chino‐Flores, C. , Dantán‐González, E. , Vázquez‐Ramos, A. , Tinoco‐Valencia, R. , Díaz‐Méndez, R. , Sánchez‐Salinas, E. , … Ortiz‐Hernández, M. L. (2012). Isolation of the opdE gene that encodes for a new hydrolase of *Enterobacter* sp. capable of degrading organophosphorus pesticides. Biodegradation, 23(3), 387–397. 22065283 10.1007/s10532-011-9517-6

[mbo3507-bib-0011] Chung, T. P. , Tseng, H. Y. , & Juang, R. S. (2003). Mass transfer effect and intermediate detection for phenol degradation in immobilized *Pseudomonas putida* systems. Process Biochemistry, 38(10), 1497–1507.

[mbo3507-bib-0012] Datta, S. , Christena, L. R. , & Rajaram, Y. R. S. (2012). Enzyme immobilization: An overview on techniques and support materials. *3* . Biotechnology, 3, 1–9. 10.1007/s13205-012-0071-7 PMC3563746 28324347

[mbo3507-bib-0013] Dereeper, A. , Audic, S. , Claverie, J.‐M. , & Blanc, G. (2010). BLAST‐EXPLORER helps you building datasets for phylogenetic analysis. BMC evolutionary biology, 10, 1–6. 20067610 10.1186/1471-2148-10-8 PMC2821324

[mbo3507-bib-0014] Dereeper, A. , Guignon, V. , Blanc, G. , Audic, S. , Buffet, S. , Chevenet, F. , … Gascuel, O. (2008). Phylogeny.fr: Robust phylogenetic analysis for the non‐specialist. Nucleic acids research, 36, 465–469. 10.1093/nar/gkn180 PMC2447785 18424797

[mbo3507-bib-0015] Ekkhunnatham, A. , Jongsareejit, B. , Yamkunthong, W. , & Wichitwechkarn, J. (2012). Purification and characterization of methyl parathion hydrolase from *Burkholderia cepacia* capable of degrading organophosphate insecticides. World Journal of Microbiology and Biotechnology, 28(4), 1739–1746. 22805956 10.1007/s11274-011-0985-y

[mbo3507-bib-0016] Ellman, G. L. , Courtney, K. D. , Andres, V. , & Featherstone, R. M. (1961). A new and rapid colorimetric determination of acetylcholinesterase activity. Biochemical Pharmacology, 7(2), 88–95. 13726518 10.1016/0006-2952(61)90145-9

[mbo3507-bib-0017] Felske, A. , Engelen, B. , Nübel, U. , & Backhaus, H. (1996). Direct ribosome isolation from soil to extract bacterial rRNA for community analysis. Applied and Environmental Microbiology, 62(11), 4162–4167. 8900007 10.1128/aem.62.11.4162-4167.1996 PMC168238

[mbo3507-bib-0018] Goda, S. K. , Elsayed, I. E. , Khodair, T. A. , El‐Sayed, W. , & Mohamed, M. E. (2010). Screening for and isolation and identification of malathion‐degrading bacteria: Cloning and sequencing a gene that potentially encodes the malathion‐degrading enzyme, carboxylestrase in soil bacteria. Biodegradation, 21(6), 903–913. 20401686 10.1007/s10532-010-9350-3

[mbo3507-bib-0019] Ismail, W. , & Gescher, J. (2012). Epoxy coenzyme a thioester pathways for degradation of aromatic compounds. Applied and Environmental Microbiology, 78(15), 5043–5051. 22582071 10.1128/AEM.00633-12 PMC3416408

[mbo3507-bib-0020] Kadakol, J. C. , Kamanavalli, C. M. , & Shouche, Y. (2011). Biodegradation of Carbofuran phenol by free and immobilized cells of Klebsiella pneumoniae ATCC13883T. World Journal of Microbiology and Biotechnology, 27(1), 25–29.

[mbo3507-bib-0021] Kadiyala, V. , & Spain, J. C. (1998). A two‐component monooxygenase catalyzes both the hydroxylation of p‐ nitrophenol and the oxidative release of nitrite from 4‐nitrocatechol in Bacillus sphaericus JS905. Applied and Environmental Microbiology, 64(7), 2479–2484. 9647818 10.1128/aem.64.7.2479-2484.1998 PMC106414

[mbo3507-bib-0022] Kawahara, K. , Tanaka, A. , Yoon, J. , & Yokota, A. (2010). Reclassification of a parathione‐degrading *Flavobacterium* sp. ATCC 27551 as *Sphingobium fuliginis* . The Journal of General and Applied Microbiology, 56(3), 249–255. 20647682 10.2323/jgam.56.249

[mbo3507-bib-0023] Kitagawa, W. , Kimura, N. , & Kamagata, Y. (2004). A novel p‐nitrophenol degradation gene cluster from a gram‐positive bacterium, *Rhodococcus opacus* SAO101. Journal of Bacteriology, 186(15), 4894–4902. 15262926 10.1128/JB.186.15.4894-4902.2004 PMC451640

[mbo3507-bib-0024] Kulkarni, M. , & Chaudhari, A. (2007). Microbial remediation of nitro‐aromatic compounds : An overview. Journal of Environmental Management, 85, 496–512. 17703873 10.1016/j.jenvman.2007.06.009

[mbo3507-bib-0025] Leung, K. T. , Moore, M. , Lee, H. , & Trevors, J. T. (2005). Effect of carbon starvation on p ‐nitrophenol degradation by a *Moraxella* strain in buffer and river water. FEMS Microbiology Ecology, 51, 237–245. 16329872 10.1016/j.femsec.2004.08.007

[mbo3507-bib-0026] Liang, Y. L. , Zhang, Z. , Wu, M. , Wu, Y. , & Feng, J. X. (2014). Isolation, screening, and identification of cellulolytic bacteria from natural reserves in the subtropical region of China and optimization of cellulase production by *Paenibacillus terrae* ME27‐1. BioMed Research International, 2014, 1–13. 10.1155/2014/512497 PMC4090499 25050355

[mbo3507-bib-0027] Lin, J. , Gan, L. , Chen, Z. , & Naidu, R . (2015). Biodegradation of tetradecane using *Acinetobacter venetianus* immobilized on bagasse. Biochemical Engineering Journal, 100, 76–82.

[mbo3507-bib-0028] Martín, M. , Mengs, G. , Plaza, E. , Garbi, C. , Sánchez, M. , Gibello, A. , … Ferrer, E. (2000). Propachlor removal by *Pseudomonas* strain GCH1 in an immobilized‐cell system. Applied and Environmental Microbiology, 66(3), 1190–1194. 10698790 10.1128/aem.66.3.1190-1194.2000 PMC91961

[mbo3507-bib-0029] Martínez‐Ocampo, F. , Beltrán, F. L.‐A. , Hernández‐mendoza, A. , Rojas‐Espinoza, L. E. , Popoca‐Ursino, E. C. , Ortiz‐Hernández, M. L. , … Dantán‐González, E. (2015). *Burkholderia cenocepacia* strain CEIB S5‐1, a rhizosphere‐inhabiting bacterium with potential in bioremediation. Genome Announcements, 3(2), 1–2. 10.1128/genomeA.00056-15 PMC4358383 25744996

[mbo3507-bib-0030] O'Reilly, K. T. , & Crawford, R. L. (1989). Degradation of pentachlorophenol by polyurethane‐immobilized Flavobacterium cells. Applied and Environmental Microbiology, 55(9), 2113–2118. 2508552 10.1128/aem.55.9.2113-2118.1989 PMC203041

[mbo3507-bib-0031] Ortiz‐Hernández, M. L. , & Sánchez‐Salinas, E. (2010). Biodegradation of the organophosphate pesticide tetrachlorvinphos by bacteria isolated from agricultural soils in Mexico. Revista Internacional de Contaminacion Ambiental, 26(1), 27–38.

[mbo3507-bib-0032] Ray, P. , Oubelli, M. A. , & Löser, C. (1999). Aerobic 4‐nitrophenol degradation by microorganisms fixed in a continuously working aerated solid‐bed reactor. Applied Microbiology Biotechnology, 51, 284–290. 10091334 10.1007/s002530051394

[mbo3507-bib-0033] Saez, J. M. , Aparicio, J. D. , Amoroso, M. J. , & Benimeli, C. S . (2015). Effect of the acclimation of a Streptomyces consortium on lindane biodegradation by free and immobilized cells. Process Biochemistry, 50, 1923–1933.

[mbo3507-bib-0034] Samuel, M. S. , Sivaramakrishna, A. , & Mehta, A. (2014). Bioremediation of p‐nitrophenol by Pseudomonas putida 1274 strain. Journal of Environmental Health Science & Engineering, 12(53), 1–8. 24581307 10.1186/2052-336X-12-53 PMC3996030

[mbo3507-bib-0035] Sethunathan, N. , & Yoshida, T. (1973). Parathion degradation in submerged rice soils in the Philippines. Journal of Agricultural and Food Chemistry, 21(3), 504–506. 4708819 10.1021/jf60187a040

[mbo3507-bib-0036] Shindo, S. , Takata, S. , Taguchi, H. , & Yoshimura, N. (2001). Development of novel carrier using natural zeolite and continuous ethanol fermentation with immobilized *Saccharomyces cerevisiae* in a bioreactor. Biotechnology Letters, 23(24), 2001–2004.

[mbo3507-bib-0037] Sogorb, M. A. , & Vilanova, E. (2002). Enzymes involved in the detoxification of organophosphorus, carbamate and pyrethroid insecticides through hydrolysis. Toxicology Letters, 128(1–3), 215–228. 11869832 10.1016/s0378-4274(01)00543-4

[mbo3507-bib-0038] Spain, J. C. , & Gibson, D. T. (1991). Pathway for biodegradation of p‐nitrophenol in a Moraxella sp. Applied and Environmental Microbiology, 57(3), 812–819. 16348446 10.1128/aem.57.3.812-819.1991 PMC182799

[mbo3507-bib-0039] Stelting, S. , Burns, R. G. , Sunna, A. , Visnovsky, G. , & Bunt, C. R. (2012). Immobilization of Pseudomonas sp. strain ADP: A stable inoculant for the bioremediation of atrazine. Applied Clay Science, 64, 90–93.

[mbo3507-bib-0040] Sultatos, L. G. (2006). Toxicology of organophosphate and carbamate compounds In Gupta Ramesh C. (Ed.), Toxicology of Organophosphate & Carbamate Compounds (pp. 209–218). Amsterdam; Boston: Elsevier Academic Press.

[mbo3507-bib-0041] Tallur, P. N. , Mulla, S. I. , Megadi, V. B. , Talwar, M. P. , & Ninnekar, H. Z. (2015). Biodegradation of cypermethrin by immobilized cells of *Micrococcus* sp. strain CPN 1. Brazilian Journal of Microbiology, 46(3), 667–672. 26413046 10.1590/S1517-838246320130557 PMC4568881

[mbo3507-bib-0042] Tamura, K. , Stecher, G. , Peterson, D. , Filipski, A. , & Kumar, S. (2013). MEGA6: Molecular evolutionary genetics analysis version 6. 0. Molecular Biology and Evolution, 30(12), 2725–2729. 24132122 10.1093/molbev/mst197 PMC3840312

[mbo3507-bib-0043] Tope, A. M. , Srinivas, N. , Kulkarni, S. J. , & Jamil, K. (2001). Mesoporous molecular sieve (MCM‐41) as support material for microbial cell immobilization and transformation of 2,4,6‐trinitrotoluene (TNT): A novel system for whole cell immobilization. Journal of Molecular Catalysis ‐ B Enzymatic, 16(1), 17–26.

[mbo3507-bib-0044] Torres, L. G. , Ramos, F. , Avila, M. A. , & Ortiz, I. (2012). Removal of methyl parathion by surfactant‐assisted soil washing and subsequent wastewater biological treatment. Journal of Pesticide Science, 37(3), 240–246.

[mbo3507-bib-0045] Vikram, S. , Pandey, J. , Bhalla, N. , Pandey, G. , Ghosh, A. , Khan, F. , … Raghava, G. P. (2012). Branching of the p‐nitrophenol (PNP) degradation pathway in *Burkholderia* sp. Strain SJ98: Evidences from genetic characterization of PNP gene cluster. AMB Express, 2(1), 30. 22681853 10.1186/2191-0855-2-30 PMC3485097

[mbo3507-bib-0046] Wang, X. , Wu, N. , Guo, J. , Chu, X. , Tian, J. , Yao, B. , & Fan, Y. (2008). Phytodegradation of organophosphorus compounds by transgenic plants expressing a bacterial organophosphorus hydrolase. Biochemical and Biophysical Research Communications, 365(3), 453–458. 17996731 10.1016/j.bbrc.2007.10.193

[mbo3507-bib-0047] WHO (2009). The WHO recommended classification of pesticides by hazard and guidelines to classification 2009 (p. 78). Wissenchaftliche Verlagsgesellschaft mbH, Stuttgart, Germany: World Health Organization.

[mbo3507-bib-0048] Wilson, J. D. , Colman, J. , & Sutton, C . (2001). Toxicological profile for methyl parathion. Atlanta: U.S. Department of Healt and Human Services, Public Health Service, Agency for Toxic Substances and Disease Registry. 38349976

[mbo3507-bib-0049] Yañez‐Ocampo, G. , Sanchez‐Salinas, E. , Jimenez‐Tobon, G. A. , Penninckx, M. , & Ortiz‐Hernández, M. L. (2009). Removal of two organophosphate pesticides by a bacterial consortium immobilized in alginate or tezontle. Journal of Hazardous Materials, 168(2–3), 1554–1561. 19362771 10.1016/j.jhazmat.2009.03.047

[mbo3507-bib-0050] Yáñez‐Ocampo, G. , Sánchez‐Salinas, E. , & Ortiz‐Hernández, M. L. (2011). Removal of methyl parathion and tetrachlorvinphos by a bacterial consortium immobilized on tezontle‐packed up‐flow reactor. Biodegradation, 22(6), 1203–1213. 21533773 10.1007/s10532-011-9475-z

[mbo3507-bib-0051] Yang, L. , Lu, M. , Carl, S. , Mayer, J. A. , Cushman, J. C. , Tian, E. , & Lin, H. (2015). Biomass characterization of Agave and Opuntia as potential biofuel feedstocks. Biomass and Bioenergy, 76, 43–53.

[mbo3507-bib-0052] Zhang, J. , Sun, Z. , Li, Y. , Peng, X. , Li, W. , & Yan, Y. (2009). Biodegradation of p ‐nitrophenol by Rhodococcus sp. CN6 with high cell surface hydrophobicity. Journal of Hazardous Materials, 163, 723–728. 18718714 10.1016/j.jhazmat.2008.07.018

[mbo3507-bib-0053] Zhang, S. , Sun, W. , Xu, L. , Zheng, X. , Chu, X. , Tian, J. , … Fan, Y. (2012). Identification of the para‐nitrophenol catabolic pathway, and characterization of three enzymes involved in the hydroquinone pathway, in *Pseudomonas* sp. 1‐7. BMC Microbiology, 12(1), 1–11. 22380602 10.1186/1471-2180-12-27 PMC3324391

[mbo3507-bib-0054] Zohar, S. , Kviatkovski, I. , & Masaphy, S. (2013). Increasing tolerance to and degradation of high p‐nitrophenol concentrations by inoculum size manipulations of Arthrobacter 4H b isolated from agricultural soil. International Biodeterioration & Biodegradation, 84, 80–85.

